# The peer effects of corporate poverty alleviation behavior: Empirical evidence from China

**DOI:** 10.1371/journal.pone.0304252

**Published:** 2024-07-15

**Authors:** Kang Fang, Li Zheng, Ningning Zhai

**Affiliations:** 1 School of Business, Qingdao University, Qingdao, China; 2 School of Economics, Nankai University, Tianjin, China; Central University of Finance and Economics, CHINA

## Abstract

This study explores the peer and economic effects of corporate poverty alleviation behavior. Using the data of A-share non-financial listed corporates in Shanghai and Shenzhen of China from 2016 to 2020, the empirical analysis of this study finds that: corporate poverty alleviation behavior has significant peer effects; the guidance of local poverty alleviation policies weakens the peer effects of corporate poverty alleviation behavior; compared to private enterprises, the poverty alleviation behavior of the peer firms has a more significant impact on state-owned enterprises; and corporate poverty alleviation behavior can result in the backflow of economic benefits and achieve the organic unity of economic and social benefits. The purpose of this paper is to explore the peer effects of corporate poverty alleviation behaviors through empirical analysis using available public data. The results of the study not only increase the motivation of corporate to participate in poverty alleviation from a peer effects perspective, but also reveal key factors for sustaining corporate poverty alleviation behaviors.

## Introduction

Poverty alleviation is an important means for corporates to enhance their value and fulfill their social responsibility. Traditional corporate poverty alleviation is primarily funded by charitable donations. According to valid statistics from the Chinese government, 90% of Chinese corporates participate in poverty alleviation activities through charitable donations [1]. Previous research has shown that the behavioral motivations for corporate participation in charitable giving for poverty alleviation have evolved from early altruistic motivations [2] to strategic motivations [3, 4], political motivations, and management self-interest motivations [5]. Poverty alleviation under different motives seeks various scarce competitive resources such as moral capital [3] and social trust for corporates [6]. However, it has also been argued that traditional charitable donations to alleviate poverty can lead to problems such as infringement of shareholders’ interests, misallocation of donated resources, and restriction of corporate freedom [7]. How should corporates take a sustainable path toward poverty alleviation in the face of this contradiction?

Poverty groups essentially belong to the BOP (Bottom of Pyramid) groups, whose large population base contains enormous purchasing potential. Focusing on this group’s development needs can help businesses not only find new opportunities for growth but also avoid the profit conundrum of an increasingly saturated existing market [8] and obtain a return on investment in the marketplaces of poverty groups. However, there are several differences between the mainstream and poverty groups market, such as lack of access to information, ambiguous legal system, and varying degrees of bureaucracy [9]. It is challenging for corporations to penetrate the market of poverty groups, and they must employ innovative ways of thinking, models, and approaches to tackle complex issues.

Previous studies demonstrate that the unique way of resource integration and opportunity identification in poverty groups market present unique challenges to corporations. Previously used business models to serve mainstream markets may prevent firms from entering the market. Therefore, business model innovation is crucial for corporations to successfully attain economic returns [10]. However, innovation activities can increase the cost burden of the business process, and the corresponding resource output and risk uncertainty can inhibit the poverty alleviation motive [11]. In this context, on the one hand, corporates tend to draw on their peers’ poverty alleviation knowledge to reduce information asymmetry, design their own optimal poverty alleviation path, and lay the groundwork for joining the market of poverty groups [12]. On the other hand, corporates will imitate the business strategies of peer firms based on the competition mechanism to expand their operation markets and keep up with the competition [13]. As a result, the poverty alleviation motives of corporations are influenced by their peer firms’ poverty alleviation behavior.

China’s poverty alleviation policy has also attracted many corporations to start poverty alleviation initiatives [1]. In the current market environment, the government still holds a dominant position in allocating important resources and tightly controlling the vetting of business activities [14, 15]. For instance, when government favoritism is reduced, financing for corporates in the area will become much more difficult compared to other places [16]. The Chinese government has released many policy documents between 2016 and 2019 to support corporates in poverty alleviation. Guided by government policies, poverty alleviation is both an effective means for enterprises to improve their legitimacy and a reliable way to obtain economic rent [17, 18], which strongly promotes the sustainability of corporate poverty alleviation behavior.

When making business decisions, corporations should concentrate on their own features in addition to the external environment. The poverty alleviation behaviors of corporations with varied property rights exhibit different business patterns, as these behaviors place a greater emphasis on social responsibility. The social responsibility index formed by the long-term evolution of enterprises will affect the implementation of poverty alleviation behavior [19]. Compared with private enterprises, state-owned enterprises pay more attention to their social responsibility index, and their public ownership form determines the importance of social benefits [20]. Additionally, state-owned enterprises exhibit the characteristics of large scale, strong capital liquidity, and robust economic health [21, 22], which have higher risk tolerance than private enterprises and are more suitable for poverty alleviation activities. Further, SOEs(State-owned Enterprises) implementing poverty alleviation activities will establish their social legitimacy more firmly in response to the institutional pressure of poverty alleviation policies [23, 24].

It can be seen that in the face of the BOP, a group of poor people with great purchasing potential, how corporations can break through the barriers of the poverty market to implement precise poverty alleviation is still a problem that has not been clearly explained by existing research. Previous studies have mostly focused on the internal and external factors of corporations to explore their motivation to implement poverty alleviation behaviors, and rarely considered the interactions among peer corporations. This paper explores the motivations of corporate poverty alleviation behaviors from a peer effects perspective, and examines the moderating effects of local policies and the nature of corporate property rights. The contributions of this study are as follows. First, it reveals the peer effects of corporate poverty alleviation behavior, fills a gap in the research of the external motivation of corporate poverty alleviation, and serves as a reference for the design of poverty alleviation paths for corporations at this stage. Simultaneously, under the influence of the peer effects, the poverty alleviation behavior of regional corporate can form a poverty alleviation synergy, thus promoting the poverty alleviation of poor groups and effectively facilitating the realisation of the Chinese government’s common prosperity. In addition, the economic return effect of corporate poverty alleviation can further form a sustainable trend of corporate poverty alleviation. This expands the research on the sustainability of CSR. In addition, this paper also focuses on the internal and external environment of corporate, showing the heterogeneity of the cohort effect of corporate poverty alleviation behavior under the complex business environment, which provides a reference for the development direction of corporate poverty alleviation in China.

In the next section, the theory of peer effects and its mechanism, which shapes corporate poverty alleviation behaviors, are introduced. Further, we present the differential peer effects regarding policy guidance and the nature of property rights. Finally, we demonstrate the beneficial effects of corporate poverty alleviation behaviors on corporate performance. The "Research Design" section describes the sample, variables, and model, and the "Empirical Analysis" section presents the study’s results. Finally, the “Research Conclusions and Implications” section discusses the main contributions, implications, and shortcomings.

### Theoretical analysis and hypothesis

#### Peer effects

The peer effects describe how corporate decision-making behavior is influenced by other corporations with the same reference, resulting in a tendency for consistent behavior [25]. Although both peer effects and herding effects refer to how a decision-making subject’s conduct is impacted by others, peer effects more clearly demonstrate decision-making behavior under the Rational Hypothesis of Economic Man [26, 27]. At the same time, the social multiplier effect generated by the same group behavior reveals that the behavioral modifications of the mimicked individuals will have a dramatic impact on the entire group, resulting in a magnifying effect [28]. This suggests that the peer impact of corporate poverty alleviation has significance for the overall effect of poverty alleviation in China.

In response to the mechanism of corporate peer effects, the legitimacy theory asserts that organizations will retain their legitimacy by creating the same comparison benchmark with external subjects, hence causing behavioral imitation [29]. According to the notion of information asymmetry, the amount of information held by individuals directly impacts the certainty of decision-making, and individuals will learn from other groups to reduce their uncertainty [30]. Similarly, under the influence of competitive mechanisms, peer pressure leads individuals to imitate the behavior of the surrounding groups, resulting in peer behavior [31].

Existing studies on the peer effects of corporates mainly focus on industries and regions. Gordon et al. (2020) [32] pointed out that the information disclosure behavior of peer firms can promote the innovation of focal firms. Someone indicated that corporates are more sensitive to the investment and financing behavior of peer firms when making investment and financing decisions [33]. Due to a large number of consumption, production, and innovation resources that have not been effectively developed in the poverty group market [34], a single industry cannot realize the overall resource utilization. Therefore, entering the poverty groups market is not exclusive to a specific industry. This study chooses to ignore the inherent industry framework and industry compartmentalization to study the peer effects of poverty alleviation behavior of regional corporates.

#### The impact of peer firms’ poverty alleviation behavior

The poverty penalty inherent in the poverty groups market also provides innate conditions for corporates to intervene [35]. Business model innovation based on this market demand gives corporates economic returns and efficiency advantages [36], while the non-negligible risk and uncertainty of the return cycle in the business process drives corporates to imitate and learn from their peers’ business behavior [37].

Peer effects theory suggests that information and competition are the main factors influencing the learning imitation effect of corporates [38]. Based on the information aspect, the reason why firms tend to imitate the poverty alleviation behavior of peer firms is that, on the one hand, there are entry barriers to the poverty groups market, and its inherent heterogeneity and unique demand characteristics [39] motivate firms to observe the behavior of peer firms before entering the poverty groups market. The decision information and poverty alleviation paths demonstrated by peer firms are excellent references for corporate business decisions. On the other hand, the distinctive economic, cultural, and institutional features of the poverty group market necessitate creative solutions from corporates [40]. The uncertainty and long-term nature of innovation encourage corporates to capture valid information about the innovation decisions of peer firms and reduce the cost of risk in the innovation process. As a result, faced with an overflow of poverty alleviation information from peer corporations, focal corporations tend to enter the poor market with the help of existing poverty alleviation pathways, forming imitation learning and thus breaking down market barriers.

The importance corporates place on their competitive position makes them pay special attention to the business behavior of their peers and react accordingly to changes [41]. Based on the current situation where the mainstream market is becoming more and more saturated, corporates that want to expand their market share often have to change the investment market. Due to competitive pressure, management will imitate the advanced decisions of peer firms and move closer to the market of poverty groups [42]. In addition, under the pressure of peer groups, corporates will change the direction of business decision-making to maintain their comparison benchmark and stabilize social legitimacy, demonstrating the peer characteristics of poverty alleviation decisions. Consequently, when confronted with the poverty alleviation initiatives of their peers, focal corporations are motivated to venture into impoverished markets as a means to preserve their competitive edge and external legitimacy. This external impetus is distinct from the pressures exerted by institutional factors, stakeholders, and other forces, which only influence the focal corporations when they operate within their specific cohort, revealing a pronounced boundary effect.

Based on the above analysis, corporates tend to learn and imitate the poverty alleviation behaviors of peer firms due to the influence of information and competition. Therefore, this study proposes the following hypothesis:

H1: The poverty alleviation behavior of corporates has peer effects.

### The impact of local policy guidance

Social embeddedness means that corporates gain a competitive advantage by understanding and integrating their local environment [39]. Corporations can receive the critical resources required for operations and develop a product and service network fit for the local area by partnering with the local government [8]. Currently, the social embedment of corporates in the poverty groups market cannot be separated from the network connection with the local government. Embedding in the local market with the guidance of the government will weaken the external barriers faced by enterprises, quickly grasp the demand characteristics and cultural attributes of poverty groups, reduce their information asymmetry, and thus accelerate the construction of the value orientation of the local market. Simultaneously, the decentralized reform of the fiscal system increases local governments’ autonomy in managing local affairs and resources. A good government-enterprise relationship gives a company a quality local reputation and enhances its legitimacy in the market of poverty groups. Therefore, following the provincial government will strengthen the embedment of local society and assist in the efficient implementation of poverty alleviation for corporates. However, it will reduce the attention of corporates on the poverty alleviation behavior of peers and instead focus more on government-guided behavior.

At the same time, the visible dividends of preferential poverty alleviation policies that alleviate financing constraints [42], enhance innovation performance [43], and reduce corporate risk [44] are highly attractive to downstream corporates that are at a competitive disadvantage in the mainstream market. Similarly, corporations in poverty groups market can break upstream firms’ blocking confinement if poverty alleviation policies lead them. Based on this, corporations are more likely to be attracted by policy dividend information to enter the poverty groups market and strengthen their core competitive advantage than peer firms’ poverty alleviation information.

Therefore, when corporates engage in poverty alleviation activities, they will be more sensitive to the local policy guidance behavior than their peer firms. The dividend information of the poverty alleviation policy will have a substitution effect on the poverty alleviation information of the peer firms. As a result, our hypothesis is as follows:

H2: The guidance of the local government’s poverty alleviation policy weakens the impact of the peer firms’ poverty alleviation behavior on the corporate.

### The impact of the nature of property rights

Poverty alleviation is an essential tool for corporates to fulfill their social responsibilities, and the extent of their social responsibility will directly impact the implementation. The reasons state-owned enterprises play a pioneering role in poverty alleviation are reflected in the following factors. First of all, poverty alleviation is a prominent manifestation of the superiority of socialism. The participation of state-owned enterprises extensively caters to the people’s core interests and lays a solid foundation for improving its legitimacy. Second, in addition to the mission of SOEs themselves, the political pressure exerted by local governments will prompt them to increase their poverty alleviation efforts and focus their attention on poverty alleviation industries [45]. Furthermore, state-owned enterprises bear policy burdens such as stabilizing local employment and can obtain local government assistance and low-interest state-owned bank credit [46]. As a result, when compared to private firms, SOEs have lesser financing constraints, meaning that economic goals are weighted less heavily than social goals in poverty alleviation. Furthermore, the public interest nature of state-owned capital may encourage SOEs to accept low-return poverty relief projects.

However, state-owned enterprises have some problems, such as poor marketability and a cumbersome decision-making process. Private enterprises are more innovative and have excellent market operation abilities [4]. Improved poverty alleviation activities can aid SOEs in increasing their enterprise value and core competitiveness. As the main force behind poverty reduction, state-owned firms prioritize social benefits in their business processes, and expanding investment in poverty alleviation is consistent with their economic aims [47].

To summarize, state-owned firms have stronger poverty alleviation motives and enforcement than private enterprises. When making business decisions, they are more likely to be influenced by the poverty alleviation behaviors of peer firms. Based on their attributes and external environmental guidance, SOEs tend to imitate the poverty alleviation behaviors of peer firms to enhance their legitimacy and competitive advantage. Therefore, our third hypothesis is as follows:

H3: Compared to private enterprises, the poverty alleviation behavior of the peer firms has a more significant impact on state-owned enterprises.

### The economic backflow effect of corporate poverty alleviation

When discussing corporate poverty alleviation, the topic of social responsibility is inextricably linked. When social responsibility is incorporated into a corporate management model, it helps to rationalize the corporate strategy, expand its valuable intangible strategic assets, and enhance its competitive advantage and financial performance [48]. Good social responsibility behavior can also generate a high-quality social image and stable public preference, creating a wide range of consumer groups for corporates [49]. The accumulation of corporate reputation capital driven by social responsibility improves the attractiveness of corporates to talents and facilitates the introduction of high-quality talents. The related reduction of human costs [50], government resource subsidies, and access to investment support [51] also promote the improvement of corporate financial performance. Thus, this study proposes the hypothesis:

H4: Corporates can obtain the backflow of economic benefits from poverty alleviation and improve their performance.

## Research design

### Sample design and data sources

Since 2016, the Shanghai and Shenzhen Stock exchanges have required listed corporates to provide detailed social responsibility information disclosures, including the disclosure of targeted poverty alleviation information, to encourage corporates to participate in poverty alleviation. At the same time, they are excluded to avoid bias due to the specific characteristics of the business model and financial structure of financial corporations. The Shanghai and Shenzhen A-share nonfinancial listed companies between 2016 and 2020 are selected as the research samples. Except for the data on the strength of local policy guidance, which is obtained from provincial government work reports, the rest of the data are obtained from the China Stock Market and Accounting Research Database (CSMAR). Furthermore, the data are treated as follows: (1) remove the ST and *ST company samples; (2) remove the samples with missing data; and (3) all continuous variables in the 1% and 99% quartiles are winsorized.

### Variable selection

(1) Poverty alleviation behavior of corporates (*Fp*)

Referring to Zhang et al. (2010) [52], the measurement was chosen to focus on whether the firm has substantial poverty alleviation behavior in the current year. A dummy variable (*Fp*) was established based on the poverty alleviation data disclosed by the listed companies. *Fp* takes the value of 1 if the enterprise has engaged in poverty alleviation behavior in the current year; otherwise, 0.

(2) Poverty alleviation behavior of peer firms (*Peer_Fp*)

Referring to Bramoullé et al. (2009) [53], for the peer effects of regional firms’ poverty alleviation behavior, the province was chosen as the boundary for the study. The poverty alleviation behavior of peer firms is defined as the mean value of the precise poverty alleviation behavior of other enterprises in the same province and the same year, except for the focus firms. The continuous variable *Peer_Fp* is set.

(3) Corporate performance (*Roa*)

Referring to Waddok and Graves (1997) [54], this study used corporate performance to measure the effect of economic backflow in the process of poverty alleviation by corporates. Since most studies have used the return on total assets (*Roa*) for the selection of corporate performance indicators, this measure was also chosen in this study.

(4) Local policy guidance strength (*Auth*)

Each province’s annual government work reports were evaluated using text analysis tools [55] to determine the guiding strength of poverty alleviation policies in each province by word frequency. In this study, the four keywords " rural," " poverty alleviation," " out of poverty," and " support" were selected from the annual government work report of each province. The sum of the keywords was used as the poverty alleviation policy strength of the province in that year.

(5) Nature of corporate property rights (*Soe*)

The nature of corporate property rights, according to Matuszak and Kabaciński (2021) [56], can have a significant impact on corporate decisions. In this study, the character of corporate property rights is measured as the nature of the real corporate controller. If the actual controller of the corporate is state-owned, *Soe* takes the value 1; otherwise, 0.

(6) Control variables

Drawing on related research [1, 32, 51], variables of corporate characteristics that may affect the poverty alleviation behavior of firms were controlled, including the capital structure of corporates (*Lev*), Corporate size (*Size*), Financing constraint (*SA*), Corporate age (*Age*) and a dummy variable for year (*Year*). The calculation methods for all variables are shown in Table 1.

**Table 1:** Variable definition table.

The variable name	Variable code	Variable definitions
Poverty alleviation behavior of corporate	*Fp*	Fp takes the value of 1 if the enterprise has poverty alleviation behavior in the current year; otherwise, 0.
Poverty alleviation behavior of peer firms	*Peer_Fp*	The mean value of the poverty alleviation behavior of other firms in the same province excluding the focal firms.
Return on assets	*Roa*	Return on total assets of the enterprise.
Local policy guidance strength	*Auth*	Search each province’s annual government work reports according to the keyword database.
Nature of corporate property rights	*Soe*	Measured by the nature of the real corporate controller.
Capital structure of corporates	*Lev*	Total liabilities of the listed company for the year divided by total assets.
Corporate size	*Size*	The natural logarithm of the total assets of the listed company for the year.
Financing constraint	*SA*	SA index of CSMAR.
Corporate age	*Age*	The number of years the listed company has been in existence.
year	*Year*	To set the dummy variables according to the sample interval of this study.

### Model design

STATA software was chosen to process the data, control for firm characteristics, and fix time effects. At the same time, to avoid endogeneity effects, the explained variables were lagged by one period compared to the explanatory variables.

To test hypothesis 1, the effect of the poverty alleviation behavior of peer firms on corporates. Set up the model with reference to the related study by Huang et al [1]:

$$Fp_{u,t}=\alpha +\beta_{1}Peer\_Fp_{u^{'},t-1}+Controls_{u,t-1}+\sum Year+\varepsilon$$ (1)

To test hypothesis 2, the effect of the strength of local policy guidance on the peer effects of corporate poverty alleviation behavior, the following model was designed.


$$Fp_{u,t}=\alpha +\beta_{1}Peer\_Fp_{u^{'},t-1}+\beta_{2}Peer\_Fp_{u^{'},t-1}\times Auth_{u,t-1}+\beta_{3}Auth_{u,t-1}+Controls_{u,t-1}+\sum Year+\varepsilon$$ (2)

To test hypothesis 3, the effect of the nature of corporate property rights on the peer effects of corporate poverty alleviation behavior, the following model was designed.


$$Fp_{u,t}=\alpha +\beta_{1}Peer\_Fp_{u^{'},t-1}+\beta_{2}Peer\_Fp_{u^{'},t-1}\times Soe_{u,t-1}+\beta_{3}Soe_{u,t-1}+Controls_{u,t-1}+\sum Year+\varepsilon$$ (3)

To test hypothesis 4, the economic backflow effect of corporate poverty alleviation behavior, the following model was designed.

$$Roa_{u,t}=\alpha +\beta_{1}FP_{u^{'},t-1}+Controls_{u,t-1}+\sum Year+\varepsilon$$ (4)

Where *Controls* represents the control variables, *u* represents the focal firms, *u*' represents the firms in the province other than the focal firm, *t* represents the date, *β* represents the variable coefficients, *α* represents the intercept term, and **ε** represents the random disturbance term.

## Empirical analysis

### Descriptive statistics and correlation analysis

Table 2 shows the results of the descriptive statistics and correlation analysis of the study variables. The mean value of the dummy variable for listed companies’ participation in poverty alleviation is 0.338, indicating that about 33.8% of listed companies are involved in the cause of poverty alleviation. The significant difference between the maximum and minimum values of the moderating variable Auth indicates that the policy guidance for poverty alleviation is uneven between regions. Correlation analysis shows a significant positive relationship between the explained and explanatory variables, which initially verifies hypotheses 1 and 4. Moreover, both moderating variables are significantly and positively related to corporate poverty alleviation behavior, indicating a close association.

**Table 2:** Results of descriptive statistics and correlation analysis.

	*Fp*	*Peer_Fp*	*Roa*	*Auth*	*Soe*	*Lev*	*Size*	*SA*	*Age*
*Fp*	1.000								
*Peer_Fp*	0.294***	1.000							
*Roa*	0.024***	0.002	1.000						
*Auth*	0.105***	0.325***	0.005	1.000					
*Soe*	0.264***	0.154***	-0.007	0.035***	1.000				
*Lev*	0.067***	0.027***	-0.713***	0.026***	0.156***	1.000			
*Size*	0.301***	0.034***	0.029***	-0.008	0.383***	0.282***	1.000		
*SA*	-0.039***	-0.097***	0.016**	-0.010	-0.154***	-0.027***	0.057***	1.000	
*Age*	0.133***	0.115***	-0.024***	-0.016*	0.262***	0.102***	0.147***	-0.862***	1.000
Mean	0.305	0.305	0.032	40.883	0.314	0.417	22.243	-3.836	18.944
Standard	0.460	0.148	0.264	18.364	0.464	0.326	1.344	0.257	5.680
Maximum	1	1	7.445	126	1	28.547	28.636	-2.112	62
Minimum	0	0.072	-29.608	12	0	0.008	16.649	-5.600	3

Note

***, **, and * represent significant at 1%, 5%, and 10%, respectively—the same as in the following table.

### Testing of peer effects of corporate poverty alleviation behavior

Since the explained variable *Fp* is a discontinuous dummy variable, logistic regression was used to process the data for the testing of hypotheses 1, 2, and 3. Column (1) of Table 3 examines the effects of control variables on corporate poverty alleviation behavior. The results found that corporate size, corporate age, and financing constraints promote corporate poverty alleviation, while corporate debt levels hinder it. Meanwhile, the significant coefficient values of the control variables indicate that the control variables selected in this study are appropriate.

**Table 3:** Regression results of the impact of peer firms’ poverty alleviation behavior on corporate.

Variables	(1)	(2)	(3)	(4)	(5)	(6)	(7)
*Peer_Fp*		8.996***		12.196***		7.395***	10.753***
		(14.13)		(10.81)		(10.84)	(9.35)
*Peer_Fp×Auth*				-0.076***			-0.080***
				(-4.36)			(-4.52)
*Peer_Fp×Soe*						2.678**	2.796**
						(2.47)	(2.57)
*Auth*			0.030***	0.048***			0.049***
			(8.36)	(6.61)			(6.74)
*Soe*					2.781***	1.305***	1.311***
					(8.49)	(3.32)	(3.30)
*Lev*	-0.704*	-0.992***	-0.824**	-1.047***	-1.049***	-1.291***	-1.353***
	(1.82)	(-2.62)	(-2.12)	(-2.72)	(-2.66)	(-3.39)	(-3.50)
*Size*	1.291***	1.242***	1.296***	1.267***	1.094***	1.058***	1.086***
	(14.71)	(16.02)	(15.20)	(16.02)	(12.47)	(13.81)	(13.91)
*SA*	3.666***	3.303***	3.773***	3.493***	3.136***	2.772***	2.979***
	(4.88)	(4.75)	(5.11)	(4.95)	(4.16)	(4.04)	(4.28)
*Age*	0.254***	0.220***	0.256***	0.228***	0.190***	0.165***	0.174***
	(7.09)	(6.74)	(7.28)	(6.88)	(5.31)	(5.09)	(5.28)
*Year*	yes	yes	yes	yes	yes	yes	yes
*_cons*	-22.864***	-23.834***	-23.672***	-25.770***	-20.174***	-20.917***	-22.903***
	(-7.88)	(-8.67)	(-8.29)	(6.88)	(-6.99)	(-7.68)	(-8.31)
*Log Likelihood*	-4928.868	-4816.933	-4896.712	-4790.372	-4787.825	-4682.497	-4655.523
*Waldχ* ^*2*^	367.69	522.30	428.06	538.27	347.92	516.37	523.12

Column (2) of Table 3 demonstrates the regression results of the peer effects of corporate poverty alleviation behavior, and the estimated coefficient of the explanatory variable *Peer_Fp* is positively significant at the 1% level. It implies that peer firms’ poverty alleviation behavior promotes the same in corporations. The dissemination and diffusion of poverty alleviation information among corporates in the peer group will drive corporates to form endogenous learning motivation and accelerate the poverty alleviation process.

Columns (3) and (4) of Table 3 examine the effect of the strength of local policy guidance, and the estimated coefficient of the explanatory variable Auth in Column (3) is positively significant at 1%, indicating that the guidance of poverty alleviation policies promotes corporate poverty alleviation. In Column (4), the interaction term *Peer_Fp×Auth* estimation coefficient has a negative significance at the 1% level, indicating that, under the guidance of poverty alleviation policies, corporate poverty alleviation tendencies mainly come from policy guidance rather than imitative learning from peer firms. Compared with the poverty alleviation information of peer firms, corporates are more sensitive to poverty alleviation policy information. They carry out poverty alleviation behaviors attracted by policy dividends, such as alleviating financing constraints and enhancing social reputation. Therefore, hypothesis 2 is verified, which means that the strength of local poverty alleviation policy guidance will weaken the impact of the peer firms’ poverty alleviation behavior on corporates.

Columns (5) and (6) of Table 3 examine the effect of the nature of corporate property rights. The results in Column (5) show that the estimated coefficient of the nature of property rights is significantly positive at the 1% level, and Column (6) shows that the estimated coefficient of the interaction term *Peer_Fp×Soe* is positively significant at the 5% level, which is consistent with the previous analysis. Compared to private enterprises, SOEs are more inclined to imitate the poverty alleviation behavior of peer firms. Compared with private enterprises’ economic goal-first business strategies, the essential attributes of SOEs lead to the consistency of their business strategies and social goals, verifying Hypothesis 3.

Column (7) of Table 3 shows the regression results for all variables, which still support hypotheses 1, 2, and 3.

### Testing of the economic backflow effect of corporate poverty alleviation

Since the explained variable *Roa* is a continuous variable, for testing hypothesis 4, OLS regression was used to process the data. Column (1) of Table 4 demonstrates the regression results of the effect of control variables on corporate performance, and column (2) demonstrates the effect of corporate poverty alleviation on corporate performance, with the estimated coefficient of the explanatory variable FP being positively significant at the 1% level. It shows that corporate poverty alleviation significantly enhances corporate performance and obtains the backflow of economic benefits. Based on the above, corporate poverty alleviation is no longer a mere charitable donation but a market-oriented, economic benefit-centered, and industrial development leveraged method of poverty alleviation. It is foreseeable that the improvement in corporate performance will significantly attract corporates to learn from and imitate the poverty alleviation behaviors of their peer firms, resulting in overall poverty alleviation effectiveness.

**Table 4:** Regression results of the impact of corporate poverty alleviation behavior on corporate performance.

Variables	(1)	(2)
*Fp*		0.011***
		(5.03)
*Lev*	-0.063***	-0.063***
	(-2.60)	(-2.61)
*Size*	0.007***	0.006**
	(2.85)	(2.45)
*SA*	0.009	0.007
	(0.53)	(0.40)
*Age*	-0.001	-0.001
	(-0.62)	(-0.74)
*Year*		
*_cons*	-0.066	-0.050
	(-0.62)	(-0.47)
*R* ^*2*^	0.069	0.068
*Waldχ* ^*2*^	143.53	170.88

### Robustness tests

#### Replace variables and regression method

(1) Replace the variables. The measurement of corporate poverty alleviation behavior was replaced with the amount of corporate precision poverty alleviation inputs (*Amou*), and the explanatory variables were simultaneously replaced with the mean value of precision poverty alleviation inputs (*Peer_Amou*) of other corporates in the same province and the same year, except for the focal firms, and the control variables were kept unchanged. (2) Replace the regression method. The regression method for hypotheses 2 and 3 was replaced with group regression. For hypothesis 2, the variable *Auth_power* was introduced to indicate the guiding strength of the local government’s poverty alleviation policy, and the mean value of the policy guiding strength was used as the boundary, which was greater than the mean value, *Auth_power* was taken as 1; instead, it was 0. Then, a group regression analysis was conducted according to the value of *Auth_power*. For hypothesis 3, group regression analysis was conducted based on the value of *Soe*.

The regression results in column (2) of Table 5 show that the estimated coefficient of *Peer_Amou* is positively significant, and hypothesis 1 is confirmed. Column (3) shows the regression results when the value of *Auth_power* is 0, and the estimated coefficient of *Peer_Amou* is not significant; however, as column (4) shows the regression results when the value of *Auth_power* is 1, the estimated coefficient of *Peer_Amou* is positively significant. Thus, hypothesis 2 can be confirmed by comparison. Column (5) shows the regression results when *Soe* is 0, and the estimated coefficient of *Peer_Amou* is insignificant, while column (6) shows the regression results when *Soe* is 1. The estimated coefficient of *Peer_Amou* is positively significant at the 1% level, and by comparison, hypothesis 3 holds. Following the regression of the sample, hypotheses 1, 2, and 3 were confirmed.

**Table 5:** Regression results after replacing variables and methods.

Variables	(1)	(2)	(3)	(4)	(5)	(6)
	Peer effects		Weak policy guidance	Strong policy guidance	Private enterprises	State-owned enterprises
	Amou					
*Peer_Amou*		0.150*	0.104	0.186**	0.095	0.240**
		(1.93)	(0.88)	(1.97)	(0.77)	(2.57)
*Lev*	0.243	0.146	0.491	-0.404	0.440	-0.291
	(0.96)	(0.43)	(0.98)	(-1.13)	(0.67)	(-1.03)
*Size*	0.494***	0.503***	0.492***	0.582***	0.677***	0.443***
	(11.83)	(9.38)	(6.30)	(9.29)	(7.49)	(6.77)
*SA*	2.110***	1.984***	1.873***	1.923***	1.381*	2.621***
	(7.61)	(6.47)	(4.94)	(3.70)	(1.77)	(7.28)
*Age*	0.085***	0.080***	0.072***	0.081***	0.052	0.118***
	(5.66)	(4.81)	(3.41)	(3.31)	(1.53)	(6.04)
*Year*	yes	yes	yes	yes	yes	yes
*_cons*	-0.691	-1.689	-1.819	-3.530	-6.847**	0.914
	(-0.44)	(-0.88)	(-0.67)	(-1.35)	(-1.99)	(0.37)
*R* ^*2*^	0.026	0.019	0.059	0.008	0.001	0.061

#### Control the characteristics of peer firms

The peer effects of corporate poverty alleviation behavior may also be caused by the similar characteristics of the peer firms [57]. Referring to the research of Leary and Roberts (2014) [58], this study further controlled a series of regional characteristic variables. It controlled the average characteristics of the peer firms in the region when verifying the peer effects of corporate poverty alleviation behavior, specifically, the mean capital structure of corporates, corporate size, SA index, and corporate age of regional peer firms. The regression results are shown in Table 6, and the conclusions of this paper remain robust.

**Table 6:** Regression results controlling for peer firm characteristics.

Variables	(1)	(2)	(3)	(4)	(5)	(6)
*Peer_Fp*		9.105***		12.412***		7.663***
		(13.42)		(10.58)		(10.51)
*Peer_Fp×Auth*				-0.082***		
				(-4.55)		
*Peer_Fp×Soe*						2.629**
						(2.39)
*Auth*			0.031***	0.050***		
			(8.54)	(6.75)		
*Soe*					2.599***	1.362***
					(8.46)	(3.39)
*Lev*	-0.743*	-1.019***	-0.865**	-1.060***	-1.072***	-1.325***
	(-1.94)	(-2.69)	(-2.22)	(-2.75)	(-2.77)	(-3.47)
*Size*	1.276***	1.257***	1.289***	1.276***	1.073***	1.076***
	(14.76)	(16.13)	(14.96)	(16.03)	(12.75)	(13.96)
*SA*	3.634***	3.402***	3.742***	3.531***	3.103***	2.903***
	(4.93)	(4.84)	(5.08)	(4.96)	(4.28)	(4.19)
*Age*	0.240***	0.222***	0.244***	0.228***	0.178***	0.168***
	(6.83)	(6.75)	(6.97)	(6.83)	(5.16)	(5.16)
*Peer_Lev*	5.055***	3.383*	5.522***	4.972***	4.322**	2.734
	(2.69)	(1.89)	(2.98)	(2.72)	(2.27)	(1.51)
*Peer_Size*	0.496	-0.854*	0.368	-0.586	0.076	-1.156**
	(1.03)	(-1.84)	(0.77)	(-1.24)	(0.16)	(-2.49)
*Peer_SA*	-5.303	-1.015	2.387	0.523	-5.277	-0.177
	(-1.02)	(-0.21)	(0.46)	(0.1)	(-1.03)	(-0.04)
*Peer_Age*	0.051	-0.015	0.381	0.008	0.025	0.021
	(0.2)	(-0.06)	(1.5)	(0.03)	(0.1)	(0.09)
*Year*	*yes*	*yes*	*yes*	*yes*	*yes*	*yes*
*_cons*	-56.56**	-9.811	-31.74	-13.12	-43.38*	2.699
	(-2.33)	(-0.43)	(-1.32)	(-0.56)	(-1.83)	(0.12)
*Log likelihood*	-4915.909	-4813.279	-4882.962	-4786.344	-4776.925	-4677.81
*Waldχ* ^*2*^	394.51	522.29	442.45	539.82	392.41	516.49

#### Propensity score matching (PSM) method

To eliminate possible endogeneity problems, this study used the propensity score matching (PSM) method to match the samples that have participated in poverty alleviation according to the nearest neighbor 1: 1. The standardized deviations of all covariates after matching were less than 10%, and most covariates did not differ significantly. The regression results are shown in Table 7. Following the regression of the sample, hypotheses 1, 2, and 4 were confirmed.

**Table 7:** Regression results after 1:1 nearest neighbor matching.

Variables	(1)	(2)	(3)	(4)	(5)	(6)	(7)
	Fp						Roa
*Fp*							0.006*
							(1.88)
*Peer_Fp*		13.94***		17.075***		12.066***	
		(5.88)		(5.68)		(6.89)	
*Peer_Fp×Auth*							
*Peer_Fp×Soe*						-0.826	
						(-0.49)	
*Auth*			0.038***	0.058***			
			(6.32)	(4.07)			
*Soe*					4.301***	3.340***	
					(3.36)	(3.40)	
*Lev*	-1.181	-1.672*	-1.265*	-1.854**	-0.232	-1.813**	0.036
	(-1.47)	(-1.93)	(-1.66)	(-2.14)	(-0.30)	(-2.48)	(1.30)
*Size*	1.089***	1.230***	1.070***	1.229***	0.532***	0.889***	-0.002
	(6.05)	(5.25)	(6.50)	(5.63)	(3.28)	(5.26)	(-0.63)
*SA*	3.729***	3.147**	3.878***	3.388***	0.880	2.897**	-0.031
	(2.97)	(2.50)	(3.32)	(2.71)	(0.85)	(2.58))	(-1.58)
*Age*	0.220***	0.206***	0.223***	0.212***	0.100	0.135**	-0.002**
	(3.10)	(2.78)	(3.51)	(2.96)	(1.58)	(2.31)	(-2.14)
*Year*	yes	yes	yes	yes	yes	yes	yes
*_cons*	-15.170**	-23.300***	-15.74***	-24.537***	-13.980**	-16.051***	-0.018
	(-2.32)	(-3.35)	(-2.62)	(-3.63)	(-2.34)	(-2.76)	(-0.17)
*Waldχ* ^*2*^	112.62	66.29	141.69	75.45	88.88	89.49	9.63

## Research conclusions and implications

### Research conclusions

If corporate poverty alleviation is only an extension of charitable donation, the massive investment of resources will eventually hinder the sustainable symbiosis between corporates and society. This study investigates the performance enhancement effect and explores the motivation of corporate poverty alleviation behavior from the perspective of peer effects. This study selects A-share nonfinancial listed companies in Shanghai and Shenzhen, China, from 2016 to 2020 as data samples for the study. The following findings are obtained from the empirical analysis: Within the same region, corporates imitate and learn from the poverty alleviation behaviors of their peer firms. At the same time, from the external policy guidance intensity and the property-right nature of internal enterprises, we reveal the differentiated influence of peer effects. It is found that the guidance of local poverty alleviation policies will prompt corporates to pay more attention to their positive influence and weaken their attention toward the poverty alleviation behavior of peer firms. Compared with private enterprises, state-owned enterprises are more willing to invest in the cause of poverty alleviation, and their poverty alleviation behavior has more substantial peer effects. This study investigates the underlying mechanisms behind focal corporations’ emulation of their peers’ poverty alleviation behaviors from informational and competitive perspectives. It further elucidates the primary motivations driving corporate social responsibility (CSR) engagement within the contemporary Chinese context. Additionally, the research demonstrates how corporations navigate the Bottom of the Pyramid (BOP) poverty market, influenced by both internal and external factors, to achieve economic returns.

### Theoretical contributions

Most previous studies on the motivation for corporate poverty alleviation have focused on the dividends from the fulfillment of corporate social responsibility such as alleviating financing constraints [59], accumulating reputational capital [60], enhancing consumer satisfaction [61], and reducing the cost of equity capital [62], or from external subjects such as government subsidy attraction [63] and media attention coverage. However, less attention has been paid to the impact of peer firms’ poverty alleviation behavior on focal firms. This study explores the poverty alleviation behavior of corporates from the novel perspective of peer effects, which expands the application boundary of peer effects theory, clarifies the mechanism of peer effects, and provides a new explanation for corporate poverty alleviation. The poverty alleviation behavior of enterprises guided by the social multiplier effect can easily be extended to the overall effect of regional poverty alleviation, which contributes to the inner connection between peer effects theory and common prosperity.

The sustainability of corporate poverty alleviation has always been an essential concern in the field of social responsibility, and companies are always expected to have a continuous drive to serve society [64]. Sustainability is the main challenge facing CSR(Corporate social responsibility) programs at this stage, and the large amount of scrutiny accompanying it has already exhausted corporates [65]. Previous studies have mainly addressed CSR sustainability in terms of managerial support [66], government monitoring and evaluation [67], and continuing leadership [68]. Based on the Chinese scenario, this study explains the economic backflow effect of corporate poverty alleviation behavior. On the one hand, the improvement of enterprise performance gives them a sustainable incentive to participate in poverty alleviation, and the investment in poverty alleviation is no longer just a mere charitable donation but becomes an upfront cost of benefit return; on the other hand, the performance improvement effect shown by the peer firms in the process of poverty alleviation will attract corporates, thus forming an overall poverty alleviation effect and prompting the government, a critical external body, to promote the sustainable poverty alleviation through corporates.

### Practical implications

1. When participating in poverty alleviation, corporates can refer to the paths of their peer firms in order to reduce risks and uncertainties. On the one hand, corporates can capture the demand characteristics of peer effects based on information about poverty alleviation and formulate targeted poverty alleviation strategies; on the other hand, in order to alleviate the competitive pressure brought by the poverty alleviation behavior of the peer firms, the enterprises can also develop similar strategies to maintain or even exceed their local market share.
2. Focusing on the economic development of poverty-stricken areas, local governments should always implement the strategic direction of investment promotion. The essence of poverty alleviation by enterprises should be the development and re-creation of poor groups, which plays a vital role in improving their economic interests. To promote the implementation of poverty alleviation by corporates, local governments are required to provide complete supporting services, act as "guides" for enterprises, help corporates embed in local communities, coordinate the acquisition of critical resources, and jointly establish a business ecosystem based on local characteristics with corporates.
3. Private enterprises have weakened peer effects of poverty alleviation behavior compared with state-owned enterprises. Based on the current trend of the national policy of common prosperity, state-owned enterprises should take up the responsibility of poverty alleviation, actively participate, and respond to the national call. Similarly, in the context that poverty alleviation behavior can enhance corporate performance, private enterprises should seize the opportunity, obtain the self-interest information of the peer group, precisely embed in the poverty groups, and achieve economic goals with the help of external resources and their innovative initiatives.
4. The primary connotation of “promoting common prosperity” is to promote fair income distribution. The corporate poverty alleviation behavior is directly linked to poverty groups, which plays a crucial role in reducing the absolute income gap and continuously promoting the downward movement of the Gini coefficient. The peer effects of corporate poverty alleviation behaviors promote the realization of the overall social poverty alleviation effect. Government departments should strengthen the degree and quality of the information disclosure on corporate poverty alleviation and publicize successful cases and their operation mechanism. At the same time, to encourage corporates to actively participate in poverty alleviation, government departments can set up poverty alleviation index indicators, continuously refine corporate social responsibility, expand the influence of poverty alleviation behaviors, and gather the general trend of common prosperity.

### Limitations and future research

1. The study only explored the internal and external influencing factors of the peer effects of corporate poverty alleviation behavior through the dual transmission chain of policy guidance and property rights nature, which is narrow in scope. Subsequent studies can be based on the implementation process of corporate poverty alleviation, that is, financing constraints, social responsibility, and manager’s pressure, to ensure the adaptability of the results.
2. The study only explored the positive effects of poverty alleviation behaviors on corporates and did not delve into the direct effects of corporate poverty alleviation behaviors on poverty groups. Since different poverty alleviation approaches bring about differentiated effects on poverty groups, subsequent studies can explore the heterogeneous effects of different poverty alleviation models of corporates on poverty groups and provide suggestions for future poverty alleviation directions of corporates.
3. This study is exclusively concerned with the peer effect of poverty alleviation behaviors among Chinese corporations, omitting multi-country research contexts. Consequently, the generalizability of the findings remains subject to discussion. Future research could explore the extent to which corporations are influenced by their peers in their poverty alleviation efforts and the economic outcomes of such behaviors, taking into account the market dynamics of poverty groups in various countries.

## Supporting information

S1 File Paper data. (XLS)

## References

[1] Huang H, Shang R, Wang L, et al. Corporate social responsibility and firmvalue: evidence from Chinese targeted poverty alleviation[J]. Management Decision, 2022, 60(12): 3255–3274. doi: 10.1108/MD-07-2021-0993

[2] Campbell L, Gulas C S, Gruca T S. Corporate giving behavior and decision-maker social consciousness[J]. Journal of Business Ethics, 1999, 19(4): 375–383. doi: 10.1023/A:1006080417909

[3] Godfrey P C. The relationship between corporate philanthropy and shareholder wealth: A risk management perspective[J]. Academy of Management Review, 2005, 30(4): 777–798. 10.5465/amr.2005.18378878.

[4] Porter M E, Kramer M. R. The competitive advantage of corporate philanthropy[J]. HarvardBusinessReview, 2002, 80(12): 56–68, 133. https://europepmc.org/article/med/12510538. 12510538

[5] Atkinson L, Galaskiewicz J. Stock ownership and company contributions to charity[J]. Administrative Science Quarterly, 1988: 82–100. 10.2307/2392856.

[6] Wang H, Qian C. “Corporate philanthropy and corporate financial performance: the roles of stakeholder response and political access” [J]. Academy of Management Journal, 2011, 54(6):1159–1181. 10.5465/amj.2009.0548.

[7] Bhandari A, Javakhadze D. “Corporate social responsibility and capital allocation efficiency” [J]. Journal of Corporate Finance, 2017, 43: 354–377. 10.1016/j.jcorpfin.2017.01.012.

[8] Prahalad C K. The Fortune at the Bottom of the Pyramid. Upper Saddle River, 2004, N:Wharton School Publishing.

[9] Prahalad C K. The Fortune at the Bottom of the Pyramid: Eradicating Poverty through Profits, 2005, N: Wharton School Publishing.

[10] Chesbrough H, Ahern S, Finn M, et al. Business models for technology in the developing world: The role of non-governmental organizations[J]. California Management Review, 48(3): 48–61. 10.2307/41166349.

[11] Shakeel J, Mardani A, Chofreh A G, et al. Anatomy of sustainable business model innovation[J]. Journal of cleaner production, 2020, 261: 121201. 10.1016/j.jclepro.2020.121201.

[12] Medina‐Muñoz R D, Medina‐Muñoz D R. Corporate social responsibility for poverty alleviation: An integrated research framework[J]. Business Ethics: A European Review, 2020, 29(1): 3–19. 10.1111/beer.12248.

[13] Chen M J. Competitor analysis and interfirm rivalry: Toward a theoretical integration. Academy of Management Review[J], 1996, 21(1): 100–134. 10.5465/amr.1996.9602161567.

[14] Durnev A. The real effects of political uncertainty: Elections and investment sensitivity to stock prices. Paris December 2010 Finance Meeting EUROFIDAI-AFFI. https://ssrn.com/abstract=1695382.

[15] Fan J P H, Wong T J, Zhang T. Politically connected CEOs, corporate governance, and Post-IPO performance of China’s newly partially privatized firms[J]. Journal of Financial Economics, 2007, 84(2): 330–357. 10.1016/j.jfineco.2006.03.008.

[16] Boubakri N, Cosset J C, Saffar W. The impact of political connections onfirms’operating performance and financing decisions[J]. Journal of FinancialResearch, 2012, 35(3): 397–423. 10.1111/j.1475-6803.2012.01322.x.

[17] Yan J Z, Chang S J. The contingent effects of political strategies on firm performance: A political network perspective[J]. Strategic Management Journal, 2018, 39(8): 2152–2177. 10.1002/smj.2908.

[18] Su J, He J. Does giving lead to getting? Evidence from Chinese private enterprises[J]. Journal of Business Ethics, 2010, 93(1): 73–90. 10.1007/s10551-009-0183-0.

[19] Chen J, Dong W, Tong J, et al. “Corporate philanthropy and investment efficiency:empirical evidence from China” [J]. Pacific-Basin Finance Journal,2018, 51: 392–409. 10.1016/j.pacfin.2018.08.008.

[20] Vollero A, Conte F, Siano A, et al. Corporate social responsibility information and involvement strategies in controversial industries[J]. Corporate Social Responsibility and Environmental Management, 2019, 26(1): 141–151. 10.1002/csr.1666.

[21] Florio M. Contemporary public enterprises: innovation, accountability, governance[J]. Journal of Economic Policy Reform, 2014, 17(3): 201–208. 10.1080/17487870.2014.913823.

[22] Tan C, Puchniak D W, Varonil, U. State-owned enterprises in Singapore: Historical insights into a potential model for reform[J]. Columbia Journal of Asian Law, 2015, 28(2): 61–97. https://heinonline.org/HOL/LandingPage?handle=hein.journals/colas28&div=10&id=&page=.

[23] Bo H, Li T, Toolsema L A. Corporate social responsibility investment and social objectives: An examination on social welfare investment of Chinese state owned enterprises[J]. Scottish Journal of Political Economy, 2009, 56(3): 267–295. 10.1111/j.1467-9485.2009.00484.x.

[24] Shi W, Hoskisson R E, Zhang Y A. A geopolitical perspective into the opposition to globalizing state‐owned enterprises in target states[J]. Global Strategy Journal, 2016, 6(1): 13–30. 10.1002/gsj.1105.

[25] Wu Z, Salomon R. Does imitation reduce the liability of foreignness? Linking distance, isomorphism, and performance[J]. Strategic Management Journal, 2016, 37(12): 2441–2462. 10.1002/smj.2462.

[26] Banerjee A V. A simple model of heard behavior[J]. The Quarterly Journal of Economics, 1992, 107(3): 797–817. 10.2307/2118364.

[27] Bikhchandani S, Hirshleifer D, Welch I. A theory of fads, fashion, custom, and cultural change as informational cascades[J]. Journal of Political Economy, 1992, 100(5): 992–1026. 10.1086/261849.

[28] Kaustia M, Rantala V. Social Learning and Corporate Peer Effects[J]. Journal of Financial Eco-nomics, 2015, 117(3): 653–669. 10.1016/j.jfineco.2015.06.006.

[29] Parsons C A, Sulaeman J, Titman S. The Geography of Financial Misconduct[J]. Journal of Finance, 2018, 73(5): 2087–2137. 10.1111/jofi.12704.

[30] Hogg M A. Uncertainty-identity theory[J]. Advances in Experimental Social Psychology, 2007, 39(6): 69–126. 10.1016/S0065-2601(06)39002-8.

[31] Brechwald W A, Prinstein M J. Beyond homophily: A decade of advances in understanding peer influence processes[J]. Journal of Research on Adolescence, 2011, 21(1): 166–179. 10.1111/j.1532-7795.2010.00721.x. 23730122 PMC3666937

[32] Gordon E A, Hsu H T, Huang H. Peer R&D disclosure and corporate innovation: Evidence from American depositary receipt firms[J]. Advances in Accounting, 2020, 49: 1–13. 10.1016/j.adiac.2020.100471.

[33] Dougal C, Parsons C A, Titman S. Urban vibrancy and corporate growth[J]. The Journal of Finance, 2015, 70(1): 163–210. 10.1111/jofi.12215.

[34] Zhou J, Xing X, Tong Y. Serving the low-income group with microfinance in China[J]. Portland International Conference on Management of Engineering & Technology, 2009: 1758–1764.https://10.1109/PICMET.2009.5261943.

[35] Hammond A L, Kramer W J, Katz R.S, et al. The next 4 billion[J]. Innovations: Technology,Governance, Globalization, 2007, 2(1–2): 147–158. http://hdl.handle.net/10986/4539.

[36] Brown J S, Hagel J. Innovation blowback: Disruptive management practices from Asia[J]. McKinsey Quarterly, 2005, 1: 35–45. https://www.mckinsey.com/capabilities/strategy-and-corporate-finance/our-insights/innovation-blowback-disruptive-management-practices-from-asia.

[37] Gyimah D, Machokoto M, Sikohi A. Peer influence on trade credit[J]. Journal of Corporate Finance, 2020, 64: 1–24. 10.1016/j.jcorpfin.2020.101685.

[38] Lieberman M B, Asaba S. Why do firms imitate each other[J]? Academy of Management Review, 2006, 31(2): 366–385. 10.5465/amr.2006.20208686.

[39] London T, Hart S L. Reinventing strategies for emerging markets: Beyond the transnational model[J]. Journal of International Business Studies, 2004, 35(5): 350–370. 10.1057/palgrave.jibs.8400099.

[40] Hart S L. Innovation, Creative Destruction and Sustainability[J]. Research-Technology Management, 2005, 48(5): 21–27. 10.1080/08956308.2005.11657334.

[41] Moreira S, Klueter T M, Tasselli S. Competition, technology licensing-in, and innovation[J]. Organization Science, 2020, 31(4): 1012–1036. 10.1287/orsc.2019.1337.

[42] Beck T, Klapper L F, Mendoza J C. The typology of partial credit guarantee funds around theworld[J]. Journal of Financial Stability, 2010, 6(1): 10–25. 10.1016/j.jfs.2008.12.003.

[43] Gao T, Wang H. Participation in targeted poverty alleviation and enterprise innovation investment: Analysis of the mediating effect test model basedon financing constraints[J]. Journal of Function Spaces, 2022, 10.1155/2022/7060462.

[44] Core J E, Hail L, Verdi R S. Mandatory disclosure quality, inside ownership, and cost of capital[J]. European Accounting Review, 2015, 24(1): 1–29. 10.1080/09638180.2014.985691.

[45] Fan J P H, Wong T J, Zhang T. Institutions and organizational structure: The case of state-owned corporate pyramid[J]. The Journal of Law, Economics, and Organization, 2013, 29(6): 1217–1252. 10.1093/jleo/ews028.

[46] See G K H. Harmonious society and Chinese CSR: Is there really a link[J]? Journal of Business Ethics, 2009, 89(1): 1–22. 10.1007/s10551-008-9981-z.

[47] Bai C E, Lu J, Tao Z. The multitask theory of state enterprise reform: Empirical evidence from China[J]. American Economic Review, 2006, 96(2): 353–357. https://www.aeaweb.org/articles?id=10.1257/000282806777212125.

[48] Surroca J, Tribó J A, Waddock S. Corporate responsibility and financial performance: The role of intangible resources[J]. Strategic Management Journal, 2010, 31(5): 463–490. 10.1002/smj.820.

[49] Lee H, Park T K, Moon H, et al. Corporate philanthropy, attitude towards corporations, and purchase intentions: A South Korea study[J]. Journal of Business Research, 2009, 62(10): 939–946. 10.1016/j.jbusres.2008.08.007.

[50] Fosu E, Yi K, Asiedu D. The effect of CSR on corporate social performance: Mediating role of corporate image, green innovation and moderating role of corporate identity[J]. Corporate Social Responsibility and Environmental Management, 2024, 31(1): 69–88. 10.1002/csr.2553.

[51] Zhao W, Ye G, Xu G, et al. CSR and long-term corporate performance: the moderating effects of government subsidies and peer firm’s CSR[J]. Sustainability, 2022, 14(9): 5543. 10.3390/su14095543.

[52] Zhang R, Rezaee Z, Zhu J. Corporate philanthropic disaster response and ownership type: Evidence from Chinese firms’ response to the Sichuan earthquake[J]. Journal of Business Ethics, 2010, 91(1): 51–63. 10.1007/s10551-009-0067-3.

[53] Bramoullé Y, Djebbari H, Fortin B. Identification of peer effects through social networks[J]. Journal of Econometrics, 2009, 150(1): 41–55. 10.1016/j.jeconom.2008.12.021.

[54] Waddock S A, Graves S B. The corporate social performance–financial performance link[J]. Strategic Management Journal, 1997, 18(4): 303–319. 10.1002/(SICI)1097-0266(199704)18:4<303::AID-SMJ869>3.0.CO;2-G.

[55] Koijen R S J, Philipson T J, Uhlig H. Financial health economics[J]. Econometrica, 2016, 84(1): 195–242. 10.3982/ECTA11182.

[56] Matuszak P, Kabaciński B. Non-commercial goals and financial performance of state-owned enterprises–some evidence from the electricity sector in the EU countries[J]. Journal of Comparative Economics, 2021, 49(4): 1068–1087. 10.1016/j.jce.2021.03.002.

[57] Adhikari B K, Agrawal A. Peer influence on payout policies[J]. Journal of Corporate Finance, 2018, 48: 615–637. 10.1016/j.jcorpfin.2017.12.010.

[58] Leary M T, Roberts M R. Do peer firms affect corporate financial policy[J]? The Journal of Finance, 2014, 69(1): 139–178. 10.1111/jofi.12094.

[59] García‐Sánchez I M, Hussain N, Martínez‐Ferrero J, et al. Impact of disclosure and assurance quality of corporate sustainability reports on access to finance[J]. Corporate Social Responsibility and Environmental Management, 2019, 26(4): 832–848. 10.1002/csr.1724.

[60] Eweje G. Strategic partnerships between MNEs and civil society: the post‐WSSD perspectives[J]. Sustainable Development, 2007, 15(1): 15–27. 10.1002/sd.295.

[61] Inoue Y, Lee S. Effects of different dimensions of corporate social responsibility on corporate financial performance in tourism-related industries[J]. Tourism Management, 2011, 32(4), 790–804. 10.1016/j.tourman.2010.06.019.

[62] Yi Y, Xie B, Zhou L, et al. Does CSR affect the cost of equity capital: Empirical evidence from the targeted poverty alleviation of listed companies in China[J]. Plos One, 2020, 15(2), e0227952. 10.1371/journal.pone.0227952. 32032381 PMC7006919

[63] Arya A, Mittendorf B. Supply chain consequences of subsidies for corporate social responsibility. Production and Operations Management, 2015, 24(8): 1346–1357. 10.1111/poms.12326.

[64] Dyllick T, Hockerts K. Beyond the business case for corporate sustainability[J]. Business Strategy and the Environment, 2002, 11(2): 130–141. 10.1002/bse.323.

[65] UNCTAD. Corporate social responsibility in global value chains: Evaluation and monitoringchallenges of small and medium sized suppliers in developing countries, 2012, New York and Geneva.

[66] Chatterjee S, Chaudhuri R, Vrontis D. Investigating the impacts of microlevel CSR activitieson firm sustainability: mediating role of CSR performance and moderating role of top management support[J]. Cross Cultural & Strategic Management. 10.1108/CCSM-12-2021-0228.

[67] Singh S, Holvoet N, Pandey V. Bridging sustainability and corporate social responsibility: Culture of monitoring and evaluation of CSR initiatives in India[J]. Sustainability, 2018, 10(7), 2353. 10.3390/su10072353.

[68] Nguyen H T, Le D M D, Ho T T M, et al. Enhancing sustainability in the contemporary model of CSR: A case of fast fashion industry in developing countries[J]. Social Responsibility Journal, 2020, 17(04): 578–591. 10.1108/SRJ-03-2019-0108.

